# *Staphylococcus lugdunensis* Septic Arthritis following Arthroscopic Anterior Cruciate Ligament Reconstruction

**DOI:** 10.1155/2020/2813134

**Published:** 2020-01-20

**Authors:** Saygin Kamaci, Yehia H. Bedeir, Christopher J. Utz, Angelo J. Colosimo

**Affiliations:** ^1^Department of Orthopaedic Surgery, Hacettepe University, Turkey; ^2^University of Cincinnati Medical Center, Cincinnati, OH, USA; ^3^Department of Orthopaedic Surgery, University of Alexandria, Egypt

## Abstract

*Summary*. We report two cases of *Staphylococcus lugdunensis* (*S. lugdunensis*) septic arthritis following arthroscopic anterior cruciate ligament (ACL) reconstruction. Both initial surgical procedures were ACL reconstruction along with simultaneous collateral ligament and meniscus procedures. Patients presented with septic arthritis three and ten weeks following the index procedure. Both patients successfully recovered with early arthroscopic irrigation, debridement, and synovial culture, in addition to long-term parenteral and oral antibiotics.*Staphylococcus lugdunensis* (*S. lugdunensis*) septic arthritis following arthroscopic anterior cruciate ligament (ACL) reconstruction. Both initial surgical procedures were ACL reconstruction along with simultaneous collateral ligament and meniscus procedures. Patients presented with septic arthritis three and ten weeks following the index procedure. Both patients successfully recovered with early arthroscopic irrigation, debridement, and synovial culture, in addition to long-term parenteral and oral antibiotics.*S. lugdunensis*) septic arthritis following arthroscopic anterior cruciate ligament (ACL) reconstruction. Both initial surgical procedures were ACL reconstruction along with simultaneous collateral ligament and meniscus procedures. Patients presented with septic arthritis three and ten weeks following the index procedure. Both patients successfully recovered with early arthroscopic irrigation, debridement, and synovial culture, in addition to long-term parenteral and oral antibiotics.

## 1. Introduction

Septic arthritis following anterior cruciate ligament (ACL) reconstruction is a rare (0.1-1.7%) but potentially devastating complication [1]. Postoperative septic arthritis has a negative impact on both short- and long-term outcomes of ACL reconstruction [2, 3]. Severe sequelae like full thickness cartilage lesions, degenerative arthritis, and osteomyelitis can be seen. Since cartilage loses more than half of its glycosaminoglycan and collagen content within seven days from the onset of infection, early diagnosis and prompt aggressive treatment are crucial to avoid potentially dramatic sequelae. The main goal of treatment is first to protect the articular cartilage, with a secondary goal of protecting the graft tissue. Serial arthroscopic joint debridement and lavage along with antibiotic treatment provides successful elimination of the infection and graft preservation [4].

Like other coagulase-negative staphylococcus species, widespread colonization of *Staphylococcus lugdunensis* (*S. lugdunensis*) in the human skin may play a role in potential dissemination. Skin and soft tissue infections and endocarditis are the most frequent manifestations of an *S. lugdunensis* infection. *S. lugdunensis* has been reported in the orthopaedic literature, associated with osteoarticular joint infections, prosthetic joint infections, osteomyelitis, infections related with fracture fixation devices, and arthroscopic ACL reconstruction [5].

We present the second report in the literature of two cases of *S. lugdunensis* septic arthritis following arthroscopic ACL reconstructions.

## 2. Case Reports

### 2.1. Case 1

**Figure 1:**
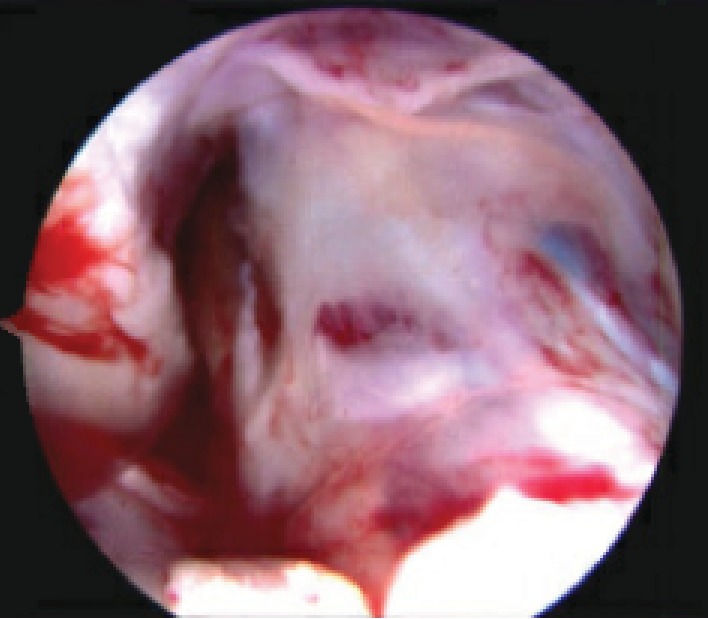
Arthroscopic picture of the ACL graft during the first irrigation and debridement.

The first case was a 20-year-old male patient who presented to the emergency department with right knee pain and increased swelling ten weeks following ACL reconstruction with a bone-patellar tendon-bone (BPTB) autograft, fibular collateral ligament (FCL) repair with augmentation with semitendinosus allograft, and a lateral partial meniscectomy. Vital signs were within normal limits. C-reactive protein (CRP) level was 26 mg/l and erythrocyte sedimentation rate (ESR) was 5 mm/h. Knee joint aspiration showed yellow, turbid aspirate consisting of 82,000/mm^3^ neutrophils. Arthroscopic knee joint irrigation, synovial debridement and synovial biopsy/culture (I&D) were performed the next day. Arthroscopy revealed an intact and well-synovialized ACL without a new meniscus tear or cartilage defect (Figure 1). The patient was admitted and intravenous ceftriaxone (2 g/day) was started. A repeat synovial biopsy/culture was performed four days later and the intraoperative culture grew *S. lugdunensis*. A hemovac drain was inserted during both procedures.

Two weeks following the arthroscopy, the knee was still swollen and blood tests revealed ESR at 43 mm/h and CRP at 59 mg/l. Knee joint aspiration showed 165,000/mm^3^ neutrophils. The FCL allograft was found to be infected, so the decision was taken to remove it. The screw hole on the lateral condyle was enlarged and the screw was loose, consistent with infection. Intraoperative cultures grew *S. lugdunensis*. Antibiotic regimen was changed to intravenous (IV) cefazolin (6 g/day) and rifampin (oral 600 mg/day) based on the result of sensitivity tests. Two repeat I&Ds were performed three and six days after the aspiration to ensure eradication of the infection. In the latter three procedures, the infected tissues seemed to be improving and a drain was not needed.

Thirty days after the initial arthroscopy, inflammatory markers dropped down to normal values (ESR 12 mm/h and CRP 2.5 mg/l). Suppression oral antibiotics continued for three months with doxycyclin (200 mg/day). Six months later, physical examination found knee flexion to be 0–130° without pain and a negative Lachman test with firm endpoint. Even though the FCL graft was removed, a varus stress test was negative, possibly due to primary repair. The patient returned back to sports with a varus-loading brace.

### 2.2. Case 2

**Figure 2:**
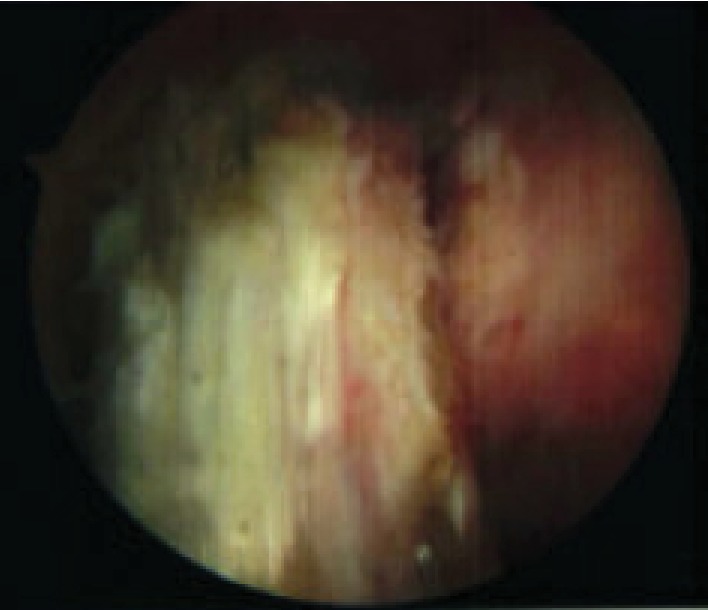
Arthroscopic picture during the initial irrigation and debridement.

**Figure 3:**
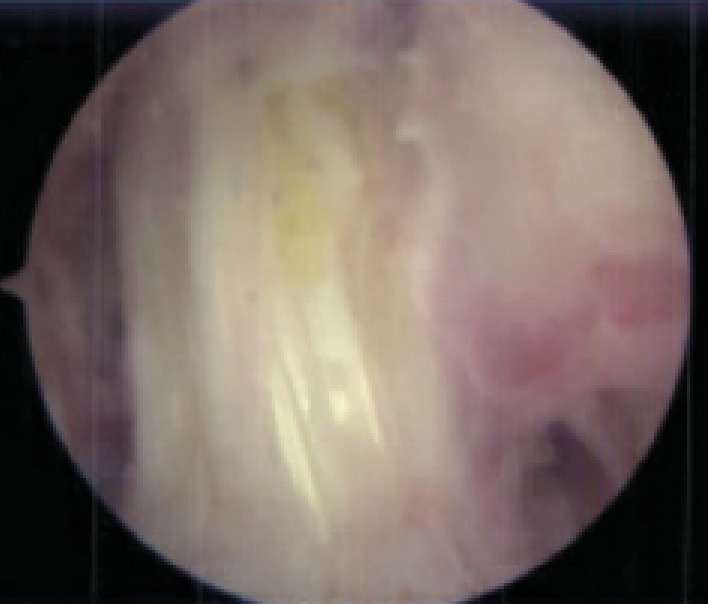
Arthroscopic picture during the third and final irrigation and debridement.

A twenty-six-year-old female presented to the office with severe right knee pain and swelling three weeks following ACL reconstruction with a hamstring autograft augmented with a semitendinosus allograft, a medial collateral ligament avulsion repair on the tibia side, all-inside medial meniscus repair, and a partial lateral meniscectomy. Vitals signs were within normal limits. Knee joint aspiration showed 40 cc of mildly turbid, yellow aspirate with intra-articular WBC at 156,750/mm^3^. Blood tests revealed ESR at 65 mm/h, CRP at 231 mg/l, and WBC at 9k. Arthroscopic I&D was performed the next day, revealing an intact ACL graft and meniscus repair without any new cartilage damage and/or new meniscus tears. IV vancomycin (2 g/day) was started immediately after surgery. Repeat I&Ds were performed at days 3 and 10 following the I&D (Figures 2 and 3). A hemovac drain was inserted in all three procedures. Cultures grew *S. lugdunensis* in three separate specimens. Cephalexin (2 g/day oral) for four weeks following IV daptomycin (8 mg/kg) for six weeks were ordered per sensitivity tests. Six weeks after treatment inflammatory markers were within normal limits. One year after treatment, the knee was symptom free, stable, and with full range of motion.

## 3. Discussion

*S. lugdunensis* was recognized as an opportunistic pathogen in 1988. The pathogenic capacity of *S. lugdunensis* puts it into a distinct position among other coagulase-negative staphylococci species. It produces a bound coagulase via a clumping factor, two adhesions and a metalloproteinase associated with deep bone and joint infections. It is a fast biofilm producer and can complete biofilm production in six hours [6]. Thus, waiting for six hours may be too late to achieve bacterial eradication with antibiotics only. Despite the low resistant rates to antimicrobial agents, biofilm production can result in persistent infections.

Conventional phenotypic identification tools using morphological and biochemical characteristics often fail to identify *S. lugdunensis*. Moreover, *S. lugdunensis* is easily misidentified as *Staphylococcus aureus* if the slide coagulase test is used rather than the tube test. Alternative molecular methods like matrix-assisted laser desorption/ionization time-of-flight mass spectrometry (MALDI TOF) is useful for identification of *S. lugdunensis* strains from other coagulase-negative staphylococci [7, 8]. The organism can still be underdiagnosed because of improper identification or dismissed as contaminants of normal skin flora in surgical biopsies. According to Sivadon et al., of the coagulase-negative staphylococci involved in bone and joint infections, *S. lugdunensis* is responsible for 7% of the cases while *Staphylococcus epidermidis* is the major isolate (81% of the cases) [9]. Thus, however, many authors believe that infections caused by this pathogen are underreported. We identified two *S. lugdunensis* infections following ACL reconstructions in the past two years.

Previously reported studies found increased operative time, tourniquet inflation time, contaminated sterile inflow cannula, contamination of the used autograft during operation, concomitant open surgical procedures, increased foreign body load, and use of drain associated with increased risk for postoperative septic arthritis following ACL reconstruction. Mei-Dan et al. published a case report in 2007 of septic arthritis with *S. lugdunensis* following ACL revision with a BPTB allograft. They underscored the history of previous ACL surgery as a potential risk factor for their case [10]. Our cases were similar and were multiligament knee injuries with additional open procedures involved in the surgery. Additional open procedures, increased surgery time, and increased foreign body load may have been risk factors in our cases.

The allografts used for the procedures were supplied by two different major tissue banks and had been cryopreserved. Although the grafts were processed with standard sterilization protocols, septic arthritis following ACL reconstruction related to contaminated tendon allografts has been published [11]. We were able to rule out the allograft as the source of infection in only one of our cases.

The relatively high virulence of *S. lugdunensis* in comparison to other coagulase-negative staphylococci, in addition to its ability to quickly form a biofilm and develop antibiotic resistance, mandates rapid and aggressive treatment. In both of our cases, we believe that early I&D in addition to long-term parenteral and oral antibiotics were key to a successful recovery.

In conclusion, *S. lugdunensis* is a coagulase-negative staphylococcus species with high virulence. Specific tools like MALDI TOF spectrometry or molecular methods are required for identification. Infection could be eradicated with quick and aggressive management including serial arthroscopic I&Ds and long-term antibiotic treatment. High suspicion should be raised in ACL infections with risk factors.
